# A comparison of femoral tunnel placement in ACL reconstruction using a 70° arthroscope through the anterolateral portal versus a 30° arthroscope through the anteromedial portal: a pilot 3D-CT study

**DOI:** 10.1186/s43019-020-00037-4

**Published:** 2020-04-03

**Authors:** Jonathan D. Kosy, Katie Walmsley, Akash D. Sharma, Elizabeth A. Gordon, Sadie V. Heddon, Rahul Anaspure, Peter J. Schranz, Vipul I. Mandalia

**Affiliations:** 1 Princess Elizabeth Orthopaedic Centre, Royal Devon and Exeter Hospital, Exeter, Devon EX2 5DW UK; 2 Hip and Knee Unit, Nuffield Orthopaedic Centre, Oxford, UK; 3 Research and Development Department, Royal Devon and Exeter Hospital, Exeter, UK; 4 Department of Musculoskeletal Radiology, Royal Devon and Exeter Hospital, Exeter, UK

**Keywords:** Anterior cruciate ligament reconstruction, Arthroscope, Femoral tunnel, Accuracy

## Abstract

**Background:**

Graft malposition is a risk factor for failure of anterior cruciate ligament reconstruction. A 70° arthroscope improves visualisation of the medial wall of the lateral femoral condyle without switching portals. We investigated whether the use of this arthroscope affected the accuracy and precision of femoral tunnel placement.

**Methods:**

Fifty consecutive adult patients were recruited. Following one withdrawal and two exclusions, 47 patients (30 in group 1 (70° arthroscope), 17 in group 2 (30° arthroscope)) underwent three-dimensional computed tomography imaging using a grid-based system to measure tunnel position.

**Results:**

No difference was found in the accuracy or precision of tunnels (mean position: group 1 = 33.3 ± 6.0% deep–shallow, 27.2 ± 5.2% high–low; group 2 = 31.7 ± 6.9% deep–shallow, 29.0 ± 6.2% high–low; not significant). A post-hoc power analysis suggests a study of 106 patients would be required.

**Conclusions:**

This pilot study suggests that tunnel position is not affected by the arthroscope used. An appropriately powered study could investigate this finding alongside other potential benefits of using a 70° arthroscope for this procedure.

**Trial registration:**

ClinicalTrials.gov, NCT02816606. Registered on 28 June 2016.

## Background

**Fig. 1:**
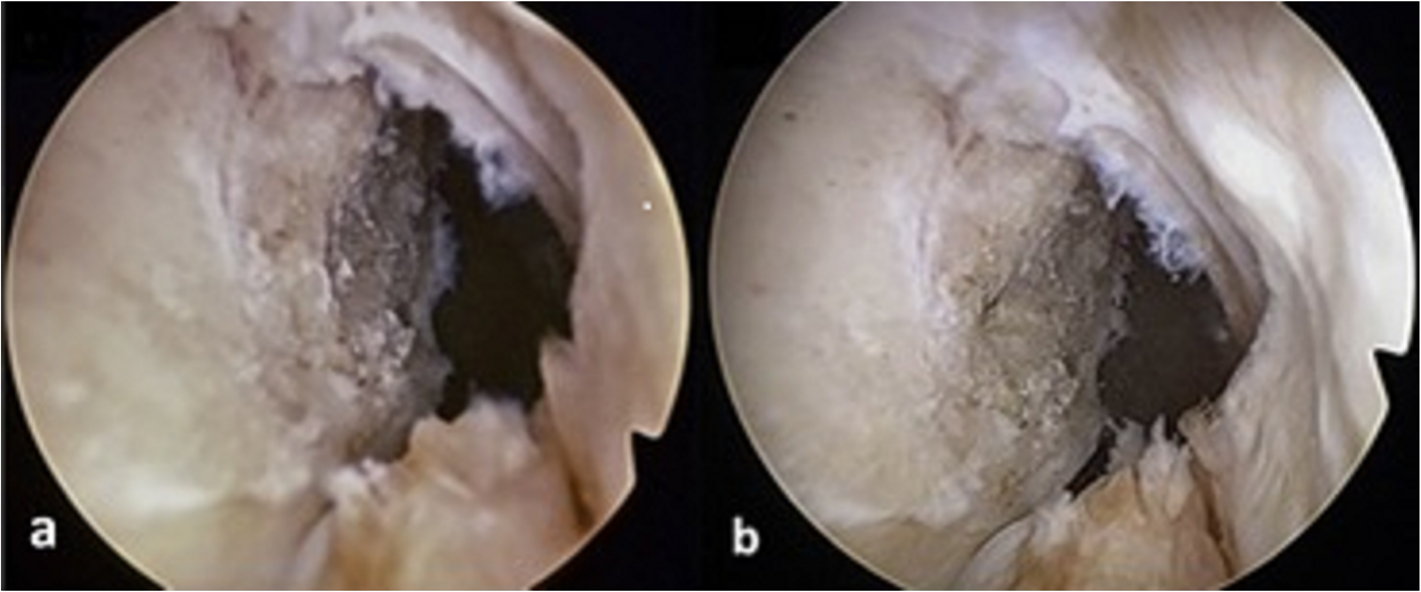
Arthroscopic view of femoral anterior cruciate ligament footprint in the right knee. **a** 30° arthroscope (via anteromedial portal); **b** 70° arthroscope (via anterolateral portal)

Graft malposition has been shown to be a major cause of failure in anterior cruciate ligament (ACL) reconstruction [1]. Therefore, a goal for the surgeon is to accurately position the tunnels in relation to either anatomical landmarks or direct measurements [2, 3]. The femoral tunnel is a particular challenge as, when the medial wall of the lateral femoral condyle is viewed from the anterolateral portal using a 30° arthroscope, a foreshortened perspective is achieved [4]. Therefore, many surgeons switch to viewing via the anteromedial portal to confirm placement (prior to tunnel drilling). However, the surgeon then must switch back to the anterolateral portal for drilling or risk over-crowding by viewing through an accessory anteromedial portal. An alternative technique is the use of a 70° arthroscope through the anterolateral portal [4, 5]. This provides a less foreshortened view of the footprint of the ACL that is comparable to the 30° arthroscope view from the anteromedial portal (Fig. 1). Perceived advantages of using the 70° arthroscope are that it prevents the need for switching between portals, it can be maintained whilst the tunnel is drilled, and it gives an improved “bird’s eye” view of the tibial footprint for tunnel placement [4, 5].

Whilst this technique is well described, no previous studies have compared the accuracy of this versus the use of a conventional 30° arthroscope. The aim of this pilot study was to directly compare the femoral tunnel positions achieved using these two arthroscopes (70° via an anterolateral portal and 30° via an anteromedial portal) in a clinical setting. Our primary hypothesis was that there would be no difference between the accuracy of the two techniques. As a pilot study, the additional goal was to inform future work comparing these techniques.

## Methods

Patients were recruited as part of a published study comparing flexible and rigid femoral tunnel reamers [6]. Ethical review board approval was additionally received, prior to study commencement, for the present analysis (study number 16/SW/0080). Informed consent for both entry into the trial and the extra imaging required was gained via consultation and a written information pack. The additional radiation exposure for one computed tomography (CT) scan (0.16 mSv) was equivalent to eight chest x-rays or 1 month’s background radiation exposure. The trial was registered with an online trial database.

Patients were randomised on the basis of the reaming system used but not the arthroscope choice. This allocation was based on surgeon preference and availability. Therefore, there were unequal numbers of patients in the study groups. No a priori power analysis was possible due to the lack of published data comparing this variable.

Fifty consecutive adult patients with an isolated ACL injury were recruited to the study. Their demographics are shown in Table 1. Patients under 18 years of age were excluded to ensure skeletal maturity and to allow the use of previously defined measurement techniques. Multi-ligament injuries and revision surgery were also excluded to prevent issues related to tunnel (and fixation) conflict. The final exclusion criterion was inadequate postoperative imaging. One patient was excluded after enrolment due to an unwillingness to attend for postoperative imaging and two patients were excluded due to incomplete posterior blow-out of the tunnel, making measurements of the tunnel aperture centre inaccurate.

**Table 1:** Patient demographics

Demographic	70° arthroscope (group 1)	30° arthroscope (group 2)
Median age in years (range)	29 (19–62)	32 (19–57)
Gender (male/female)	19/11	12/5
Side (right/left)	15/15	10/7
Total	30	17

In total, there were 31 procedures performed using a 70° arthroscope (group 1) and 18 procedures performed with the 30° arthroscope (group 2) for visualisation. One patient from each group was excluded due to incomplete posterior blow-out preventing accurate assessment of the tunnel centre.

All procedures were performed by the two senior authors (PJS and VIM, both with over 8 years of consultant experience specialising in soft-tissue knee reconstruction). The femoral tunnels were positioned under direct vision (with either arthroscope) using a microfracture awl. For group 1, all viewing was performed via the 70° arthroscope via the anterolateral portal. For group 2, the position of the intended tunnel was first marked, whilst viewing from the anterolateral portal, and then checked (and adjusted) via anteromedial portal viewing. It is routine for our surgeons to mark the tunnel in this way (first via the anterolateral tunnel) using the microfracture awl via the anteromedial portal. However, the acceptance of the tunnel position was made only after verification following switching of the portals. If required, this process was repeated until the intended tunnel position was achieved. Once accepted, a guide wire and cannulated reamer were used to create the tunnel in this position. Each surgeon used both arthroscopic techniques (in approximately equal numbers) throughout the study to avoid this being a potential source of bias to the results. Both surgeons aimed to position the graft within the anteromedial bundle position based on anatomical markers (posterior to the bifurcate ridge and inferior to the intercondylar ridge) and distance from the posterior wall. This position has been shown to be associated with a reduced incidence of graft rupture compared to a mid-bundle position [7]. All patients were reconstructed using autologous hamstring tendon graft with suspensory fixation in the femur. Where additional meniscal or chondral pathology was encountered, this was treated using the same arthroscope as used to position the femoral tunnel. Numbers were too small to compare directly these additional procedures.

**Fig. 2:**
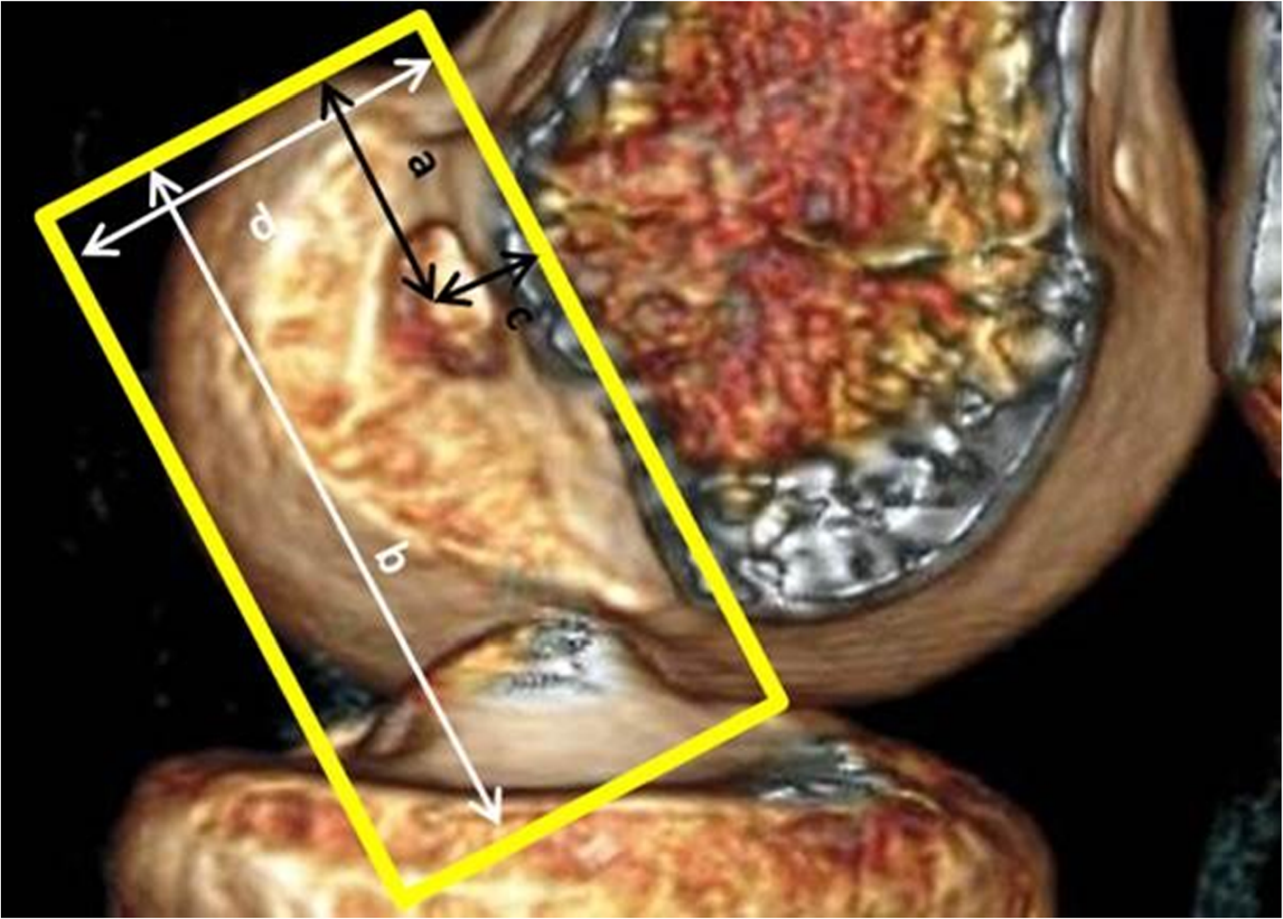
Tunnel position calculation. Three-dimensional computed tomography reconstruction image in true lateral position with digital subtraction of medial femoral condyle. Deep–shallow position = a/b; high–low position = c/d

Three-dimensional CT (3D-CT) was performed between 3 and 6 months postoperatively. Images were collected using a spiral sequence with 0.625-mm cuts. Surface rendering was applied (Advantage Workstation VolumeShare 7, GE, Waulesha, USA) with manipulation to a standardised position; the medial and lateral femoral epicondyles were overlapped (true lateral position) and the medial condyle was subtracted (in the sagittal plane through the femoral intercondylar notch). Tunnel position measurements were then made using a grid positioned along Blumensaat’s line including the maximal dimensions of the lateral condyle (Fig. 2). This method has been shown to be reliable and valid in previous studies [8, 9]. All measurements were made by the same consultant musculoskeletal radiologist who was blinded to the surgical technique used. Measurements were recorded from the centre of the aperture of the tunnel to the maximal extents of the grid using the conventional deep–shallow (DS) and high–low (HL) measurements and expressed as percentages of the total grid dimensions (Fig. 2).

**Fig. 3:**
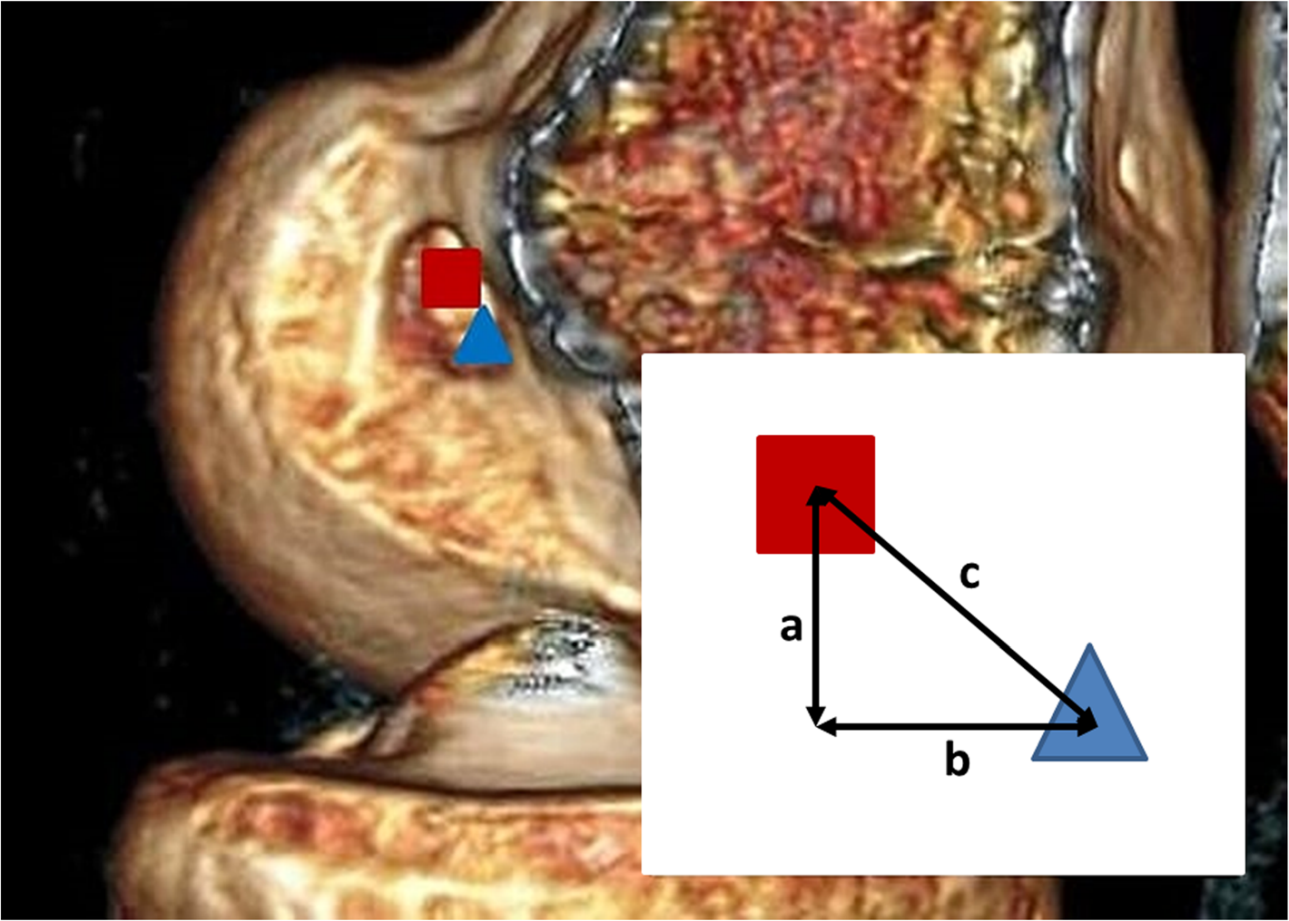
Calculation of difference between centre of tunnel aperture (AC; red square) and anatomical centre of anteromedial bundle (TC; blue triangle) shown on three-dimensional computed tomography reconstructed image; measurements a and b parallel and perpendicular (respectively) to Blumensaat’s line (on expanded view, distance c = √(a^2^ + b^2^))

The accuracy of the tunnel position was calculated in comparison to previously published data defining the anatomical position of the centre of the anteromedial bundle of the ACL using 3D-CT of cadaveric specimens (34.2% (DS); 26.3% (HL)) [10]. This point was defined as the true centre (TC). Trigonometric calculation was then used to define the distance of the aperture centre (AC) of the created tunnel to the defined optimum anatomical position (Fig. 3). The distance was calculated using the following formula: c = √(a^2^ + b^2^). The precision of the tunnel position was compared using the standard deviation of the absolute differences recorded. This methodology has previously been used in similar studies [11, 12].

Statistical analysis was performed using SPSS (version 5, IBM, Portsmouth, UK). Continuous data were compared using an unpaired *t* test. Discreet data were compared using a chi-squared test (with Yate’s correction for small numbers). Precision data were compared using Levene’s test of variance equivalence. Statistical significance was set at *P* < 0.05. A post-hoc power analysis was performed based on the calculated mean and standard deviations generated by the pilot study (α = 0.05, β = 0.8).

## Results

A summary of the results is shown in Table 2. All operated patients attended for postoperative imaging. As mentioned in the methodology, two patients were excluded from the analysis due to the presence of incomplete posterior blow-out of the tunnel (one in each group). Both blow-outs involved only the distal part of the tunnel, affecting the accurate calculation of the tunnel centre (without compromising the suspensory fixation used).

**Table 2:** Summary of results

	70° arthroscope view from anterolateral portal (Group 1)	30° arthroscope view from anteromedial portal (Group 2)	***P*** value
Position			
Depth (mean % DS ± SD)	33.3 ± 6.0	31.7 ± 6.9	n.s.
Height (mean % HL ± SD)	27.2 ± 5.2	29.0 ± 6.2	n.s.
Accuracy (% distance from TC to AC)	7.0	9.0	n.s.
Number (%) within 5%	10 (33%)	3 (18%)	n.s
Number (%) within 10%	23 (77%)	9 (53%)	n.s.
Number (%) within 15%	29 (97%)	16 (94%)	n.s.
Precision (SD of % absolute differences)	3.7	3.8	n.s.

*AC* aperture centre, *DS* deep–shallow, *HL* high–low, *n.s.* not significant, *SD* standard deviation, *TC* true centre

**Fig. 4:**
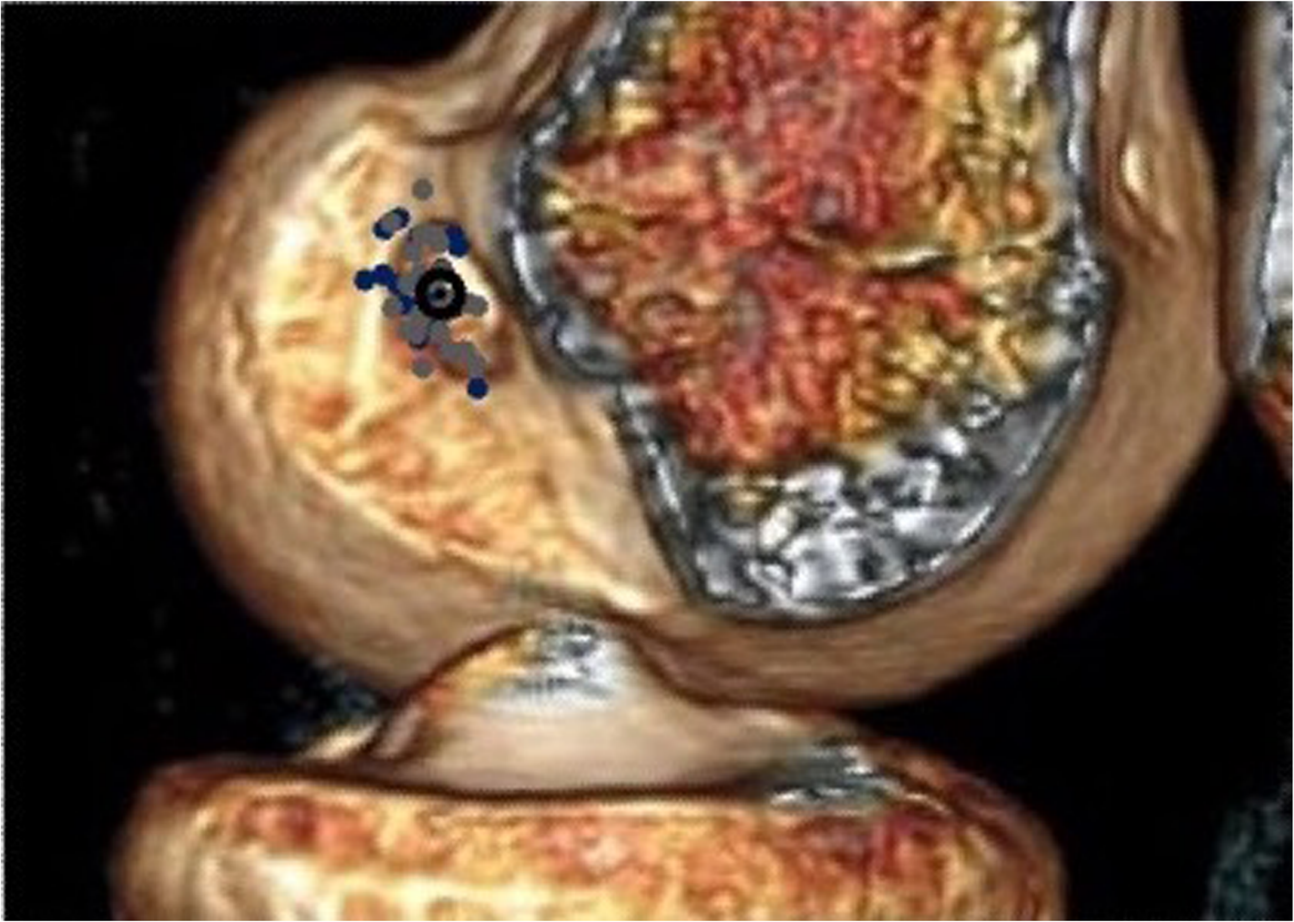
Tunnel aperture centres. Individual tunnel centres shown on three-dimensional computed tomography reconstructed image (blue dots = group 1, grey dots = group 2, black circle = anatomical centre)

In the remaining 47 patients, there was no significant difference in the position of the tunnels created or the accuracy of placement (distance between TC and AC). When the number of tunnels within 5, 10, and 15% of the absolute dimensions were compared, no difference was demonstrated. There was also no difference in the precision of the two techniques. A graphical display of the tunnel positions is shown in Fig. 4.

Post-hoc power analysis showed that a study with 106 patients (53 in each group) would be required to adequately compare the accuracy of these techniques.

## Discussion

The main finding of the study was that there was no difference between arthroscopes in centring a femoral tunnel in a desired position. With equal accuracy and precision, the variation in tunnel position likely reflects the anatomical variation seen in previous studies. The primary hypothesis would therefore have been accepted although a larger study size is required to confidently do so. It is, however, likely that a 70° arthroscope represents a worthwhile alternative to a 30° arthroscope without the additional need for portal switching. We believe this is a technique, with additional benefits, that can be readily adopted. Furthermore, this study informs continued work in this area.

Positioning of the femoral tunnel in ACL reconstruction requires good intra-operative visualisation of either native anatomy or intra-operative measurements. We believe that it is better for the surgeon to individualise tunnel position based on anatomy, but it still remains controversial exactly how the footprint should be identified and where within this the tunnel should be centred [13–15]. Our preferred choice of an anteromedial bundle position is increasingly supported by the literature with recent work demonstrating a four-fold reduction in graft failure compared to a mid-bundle position [7].

Quantitative comparison of the accuracy and precision of anatomical tunnel placement is difficult given an accepted variation in both the position of anatomical landmarks and reproducibility of surgical techniques as well as the focus of this study (the arthroscopic visualisation achieved). However, the present study used 3D-CT (shown to be the most accurate method of tunnel measurement) to compare a single defined reconstruction technique [8].

Multiple anatomical and radiological studies suggest that the ACL footprint varies in both position and size [2, 16–18] with Luites and Verdonschot reporting variation of 4 mm (in both DS and HL dimensions) about a mean value [19]. Similarly, the accuracy of surgical placement is seen to vary about a planned point. In a study by Hart et al., four different “anatomical” techniques were compared with a mean error in placement of 3–4 mm with each method [20]. Thus, there is variation in both anatomy and surgical accuracy (both in the region of 4 mm) equating to combined errors that may be relatively large (8 mm). While our results reflect these variations (in both anatomy and surgical precision) no difference was seen between the two tested arthroscopes, suggesting visualisation is equivalent.

Alternative proposed techniques (to maximise visualisation) include the use a 30° arthroscope via an accessory anteromedial portal [21, 22] or central viewing portal [23]. We chose to use the 70° arthroscope as it allows for medial portal drilling without over-crowding or additional portal creation, with most surgeons accustomed to using this arthroscope for posterior cruciate reconstruction [4, 5]. Additional benefits have also been described including the improved bird’s-eye view of the tibial footprint during tunnel positioning [5]. We found any required meniscal and chondral work also relatively easy with the 70° arthroscope, although in a less experienced group a greater learning curve may be expected (as seen with other ACL techniques) [24, 25].

Limitations of this study include those inherent to the pilot study design and the methodology used. This study was powered to show a difference in an independent variable (reamer design). The post-hoc calculation suggests that a larger study group may be required to find a difference in tunnel position between these two arthroscopic techniques. The groups were not randomised (and arthroscope choice was largely based on surgeon preference), although the demographics were well matched and the analysis was independent of knee size. The inherent potential for bias is balanced against the pragmatism of such a study. Additionally, the methodology used to define accuracy was based on previous anatomical work that may not reflect every surgeon’s preferred position. An alternative would be to study the ability to position a marker on a pre-defined goal anatomical position (for example, on the bifurcate ridge). This may give a better measure of accuracy and precision. However, conducting such a study would be problematic, with additional pre-operative imaging required outside of a cadaveric setting. The inclusion of two surgeons introduces potential bias in differences of intended tunnel position. Despite both surgeons using the two techniques in roughly equal numbers, as this was not randomised there may be inherent differences between the surgeons that may not be present in a single surgeon series. Finally, the relationship between accurate tunnel position and function or complications could not be investigated. This would require an adequately powered study solely focused on this factor to make valid conclusions. The conclusions from the present study, and recommendations made, are purposefully reserved in light of these limitations.

## Conclusion

Both arthroscopic techniques show the ability to position the femoral tunnel in an acceptable position in a clinical group. No difference was found between the techniques reported, suggesting that use of the 70° arthroscope represents a valid alternative that is worthy of consideration given the other described benefits. The importance of accuracy and precision of these techniques could be studied in a larger group with further work focussed on the impact of surgical experience and the functional consequences of potential differences identified. Furthermore, if our findings of equivalence are shown, future work should focus on the other proposed advantages of this technique including tibial tunnel visualisation as well as operative time and ease.

## Data Availability

The datasets used and/or analysed during the current study are available from the corresponding author on reasonable request.

## Abbreviations

3D-CT: Three-dimensional computed tomography
AC: Aperture centre
ACL: Anterior cruciate ligament
CT: Computed tomography
DS: Deep–shallow
HL: High–low
TC: True centre
